# The Docosanoid Neuroprotectin D1 Induces TH-Positive Neuronal Survival in a Cellular Model of Parkinson’s Disease

**DOI:** 10.1007/s10571-015-0206-6

**Published:** 2015-06-06

**Authors:** Jorgelina M. Calandria, Michelle W. Sharp, Nicolas G. Bazan

**Affiliations:** Neuroscience Center of Excellence, School of Medicine, Louisiana State University Health Sciences Center, 2020 Gravier Street, Suite D, New Orleans, LA 70112 USA

**Keywords:** Neuroprotectin D1, TH-positive neurons, Parkinson’s disease, Neuroprotection, MPP+

## Abstract

Parkinson’s disease (PD) does not manifest clinically until 80 % of striatal dopamine is reduced, thus most neuronal damage takes place before the patient presents clinical symptoms. Therefore, it is important to develop preventive strategies for this disease. In the experimental models of PD, 1-methyl-4-phenylpyridinium ion (MPP+) and rotenone induce toxicity in dopaminergic neurons. Neuroprotectin D1 (NPD1) displays neuroprotection in cells undergoing proteotoxic and oxidative stress. In the present report, we established an in vitro model using a primary neuronal culture from mesencephalic embryonic mouse tissue in which 17–20 % of neurons were TH-positive when differentiated in vitro. NPD1 (100 nM) rescued cells from apoptosis induced by MPP+ and rotenone, and the dendritic arbor of surviving neurons was examined using Sholl analysis. Rotenone, as well as MPP+ and its precursor 1-methyl-4-phenyl-1,2,3,6-tetrahydropyridine (MPTP), severely promoted retraction of dendritic arbor distal segments, thus decreasing the maximum branch order reached. On average, NPD1 counteracted the effects of MPP+ on the dendritic arborization, but failed to do so in the rotenone-treated neurons. However, rotenone did decrease the Sholl intersection number from radii 25 to 125 µm, and NPD1 did restore the pattern to control levels. Similarly, NPD1 partially reverted the dendrite retraction caused by MPP+ and MPTP. These results suggest that the apoptosis occurring in mesencephalic TH-positive neurons is not a direct consequence of mitochondrial dysfunction alone and that NPD1 signaling may be counteracting this damage. These findings lay the groundwork for the use of the in vitro model developed for future studies and for the search of specific molecular events that NPD1 targets to prevent early neurodegeneration in PD.

**Electronic supplementary material:**

The online version of this article (doi:10.1007/s10571-015-0206-6) contains supplementary material, which is available to authorized users.

## Introduction

Parkinson’s disease (PD) does not manifest clinically until 80 % of the striatal dopamine has been lost (Betarbet et al. 2002). Therefore, it is vital to develop strategies that prevent the loss of dopamine neurons.

Until recently, the significance of the selective enrichment of omega-3 essential fatty acids (docosahexaenoyl—DHA—chains of membrane phospholipids, 22C and 6 double bonds) in the nervous system had remained incompletely understood (Bazan et al. 2011; Bazan 2014). We now know that an integral membrane protein is engaged in DHA retention in photoreceptors/retinal pigment epithelial cells as a necessary event for the function of these cells (Rice et al. 2015). By studying early mechanisms of cell survival, we found that a docosanoid synthesized from docosahexaenoic acid (DHA) by 15-lipoxygenase-1 (15-LOX-1), neuroprotectin D1 (NPD1; 10*R*,17*S*-dihydroxy-docosa-4*Z*,7*Z*,11*E*,13*E*,15*E*,19*Z* hexaenoic acid) (Bazan et al. 2010; Calandria et al. 2009; Serhan et al. 2015), displays neuroprotective bioactivity. Moreover, NPD1 prevents apoptosis induced by oxidative stress (Calandria et al. 2009, 2012) involving transcriptional regulation (Calandria et al. 2015). Previously, it has been shown in experimental models of PD that MPP+ and rotenone inhibit the Complex I of the mitochondrial electron chain and causes specific toxicity to dopaminergic neurons in the substantia nigra pars compacta (SNpc) (Betarbet et al. 2000; Davis et al. 1979; Langston et al. 1983; Richardson et al. 2005).

The goal of this study was to develop an in vitro model of PD where lipid mediators could be studied in a direct manner and to determine the ability of NPD1 to enhance survival during early toxicity in mesencephalic TH-positive neurons. We used a onetime application of 1-methyl-4-phenylpyridinium ion (MPP+), 1-methyl-4-phenyl-1,2,3,6-tetrahydropyridine (MPTP), or rotenone, measuring survival and dendritic architecture modification in the remaining neurons after 24 h. In this manner, we established a cellular model to reenact the first toxic event that dopaminergic cells undergo as an early response in neurodegeneration.

## Materials and Methods

### Animals and Reagents

Animals were handled following protocols on animal experimentation approved by the Institutional Animal Care and Use Committee (IACUC), Louisiana State University Health Sciences Center (LSUHSC), New Orleans. Culture media, additives and other related reagents, including trypsin and B-27/N2, were purchased from Life Technologies (Carlsbad, CA). Fetal bovine serum (FBS) was acquired from Tissue Culture Biologicals (Long Beach, CA), and glial cell line-derived neurotrophic factor (GDNF) and transforming growth factor beta (TGF-β) were from Cell Sciences (Canton, MA). DNAseI, dbcAMP, MPTP, MPP+ and rotenone were obtained from Sigma (St. Louis, MO). Anti-tyrosine hydroxylase antibody was purchased from Pel-Freez (Rogers, AR); anti-β-tubulin III was from Sigma (St. Louis, MO) and anti-Map2 from Millipore (Billerica, MA). Secondary antibodies conjugated with Alexa Fluor 488 and 555 and nuclei staining DAPI were obtained from Life Technologies (Carlsbad, CA).

### Rat Mesencephalic TH-Positive Neurons: Primary Culture, Treatment, and Immunocytochemistry

The cultures were performed following the methods described previously (Sun et al. 2004) with some modifications. Briefly, 1 mm^3^ of the ventricular mesencephalic region was obtained from Sprague–Dawley rat embryos (embryonic day 15). Two incisions were made, one in the forebrain/mesencephalic limit and the other between the mesencephalon and the hindbrain. Once dissected from the meninges, the tissue was disaggregated using trypsin and DNaseI. Twenty thousand cells were plated in each well of an 8-well chamber slide with B-27/N2 medium containing 2.5 % serum. GDNF, TGF-β, and dcAMP were added once to increase TH-positive cells (Supplementary Fig. 1) and the media were changed every 2 days. The percentage of TH-positive cells achieved was 17–20 %. NPD1 (100 nM) was added to the culture at 7 DIV, 5 min before the addition of 100 nM rotenone, 100 µM MPP+ or 100 µM MPTP. To decide the time of exposure to MPTP and rotenone, 24- and 48-h time points were used (Supplementary Figs. 2, 3). Immunostaining was performed following a previously described protocol (Calandria et al. 2012). Briefly, cells were fixed with 4 % paraformaldehyde, permeabilized with 1 % Triton and 1 % normal serum in phosphate buffered saline (PBS) for 5 min, and then blocked with 10 % normal serum solution in PBS. The slides were incubated with the primary antibody overnight and then for 1 h with Alexa fluor-conjugated secondary antibodies.

### Image Acquisition, Assessment of the Dendritic Arbor, and Statistics

Images were obtained using a custom-built deconvolution microscope and Slidebook 4.0 software (3i-Intelligent Imaging Innovations, Inc., Denver, CO). Images were imported and analyzed using the software Imaris 7.4 (Bitplane Scientific Software, Ireland, UK) or NeuronJ (Meijering et al. 2004) plugged into ImageJ (Rasband 1997–2014). The intersection frequencies of Sholl analysis were plotted and analyzed statistically to fit a polynomial regression to assess the statistically significant differences between treatments within an experiment (Supplementary Table 1; Fig. 2q). The Sholl values were used to construct a frequency histogram and compared between treatments within each class interval and between intervals using Student’s *t* test (Tables 1, 2). The statistical analysis was performed using the SAS software (SAS Institute, Cary, NC).

**Table 1:** Sholl intersection number mean under least squares model

Radius (µm)	Treatment	Sholl least squares mean	Standard error of the least squares mean	Comparison with the control	Comparison between ± NPD1
0–25 class interval 1	Control	3.83666667	0.07223875		
	DMSO	4.24888889	0.07223875	0.0263	
	MPP^+^	2.61000000	0.07223875	<0.0001	<0.0001
	MPP^+^ + NPD1	3.61272727	0.07223875	0.9678	
	MPTP	3.59000000	0.07223875	0.8977	0.0003
	MPTP + NPD1	4.10909091	0.07223875	0.7397	
26–50 class interval 2	Control	5.06333333	0.07223875		
	DMSO	4.35111111	0.07223875	<0.0001	
	MPP^+^	1.56000000	0.07223875	<0.0001	<0.0001
	MPP^+^ + NPD1	4.02727273	0.07223875	<0.0001	
	MPTP	2.26500000	0.07223875	<0.0001	<0.0001
	MPTP + NPD1	3.70181818	0.07223875	<0.0001	
51–75 class interval 3	Control	4.62847222	0.07372836		
	DMSO	4.10666667	0.07223875	0.0003	
	MPP^+^	0.96000000	0.07223875	<0.0001	<0.0001
	MPP^+^ + NPD1	3.25090909	0.07223875	<0.0001	
	MPTP	1.04000000	0.07223875	<0.0001	<0.0001
	MPTP + NPD1	2.71636364	0.07223875	<0.0001	
76–100 class interval 4	Control	3.28000000	0.07223875		
	DMSO	2.45333333	0.07223875	<0.0001	
	MPP^+^	0.59000000	0.07223875	<0.0001	<0.0001
	MPP^+^ + NPD1	1.75090909	0.07223875	<0.0001	
	MPTP	0.55000000	0.07223875	<0.0001	<0.0001
	MPTP + NPD1	1.69818182	0.07223875	<0.0001	
100–125 class interval 5	Control	2.19333333	0.07223875		
	DMSO	0.92444444	0.07223875	<0.0001	
	MPP^+^	0.27000000	0.07223875	<0.0001	<0.0001
	MPP^+^ + NPD1	0.94363636	0.07223875	<0.0001	
	MPTP	0.37500000	0.07223875	<0.0001	0.0129
	MPTP + NPD1	0.80727273	0.07223875	<0.0001	
126–150 class interval 6	Control	0.73333333	0.07223875		
	DMSO	0.21333333	0.07223875	0.0003	
	MPP^+^	0.12500000	0.07223875	<0.0001	0.9284
	MPP^+^ + NPD1	0.36363636	0.07223875	<0.0001	
	MPTP	0.34500000	0.07223875	<0.0001	1.000
	MPTP + NPD1	0.44727273	0.07223875	<0.0001	

Mesencephalic neurons were treated with MPTP and MPP+ in the presence or absence of NPD1. The Sholl intersections numbers are shown for TH-positive neurons that survived the 24-h treatment. Data were broken down into 6 class intervals of 25-µm radius (left column) and the mean and standard error of the mean were fitted to the least squares regression model (columns 3, 4). The Scholl least squares mean values were compared pairwise against the control (column 5) and between samples in the presence or absence of NPD1 (column 6)

**Table 2:** Sholl intersection number mean under least squares model

Radius (µm)	Treatment	Sholl mean	Standard error of the mean	Sholl least squares mean	Standard error of the least squares mean	Comparison with control
0–25 class Interval 1	Control	3.566087	0.050271	3.56608696	0.05458508	
	Rotenone + NPD1	3.053846	0.051608	3.17600000	0.06257759	0.0006
	Rotenone	3.176552	0.054958	3.17655172	0.06874702	0.0021
	Rotenone + isomer	3.069712	0.04671	3.24434783	0.05458508	0.0061
26–50 class Interval 2	Control	3.655652	0.048947	3.65565217	0.05458508	
	Rotenone + NPD1	3.889143	0.07469	3.88914286	0.06257759	0.4237
	Rotenone	2.732414	0.07011	2.73241379	0.06874702	<0.0001
	Rotenone + isomer	2.709135	0.053452	2.74869565	0.05458508	<0.0001
51–75 class Interval 3	Control	3.153043	0.059304	3.15304348	0.05458508	
	Rotenone + NPD1	3.245714	0.08262	3.24571429	0.06257759	1.0000
	Rotenone	1.964138	0.066669	1.96413793	0.06874702	<0.0001
	Rotenone + isomer	1.860833	0.050282	1.89130435	0.05458508	<0.0001
76–100 class Interval 4	Control	2.726957	0.066676	2.72695652	0.05458508	
	Rotenone + NPD1	2.745143	0.080876	2.74514286	0.06257759	1.0000
	Rotenone	1.445517	0.060717	1.44551724	0.06874702	<0.0001
	Rotenone + isomer	1.236667	0.049299	1.25739130	0.05458508	<0.0001
100–125 class Interval 5	Control	2.222609	0.066566	2.22260870	0.05458508	
	Rotenone + NPD1	1.816	0.058925	1.81600000	0.06257759	<0.0001
	Rotenone	1.157241	0.071509	1.15724138	0.06874702	<0.0001
	Rotenone + isomer	0.814167	0.038327	0.82782609	0.05458508	<0.0001
126–150 class Interval 6	Control	1.848696	0.064044	1.84869565	0.05458508	
	Rotenone + NPD1	1.313143	0.057406	1.31314286	0.06257759	<0.0001
	Rotenone	0.982069	0.06284	0.98206897	0.06874702	<0.0001
	Rotenone + isomer	0.648333	0.034167	0.66173913	0.05458508	<0.0001

Mesencephalic neurons were treated with 100 nM rotenone in the presence or absence of NPD1. The Sholl intersections numbers are shown for TH-positive neurons that survived the 24-h treatment. Data were broken down into 6 class intervals of 25-µm radius (left column) and the mean and standard error of the mean (columns 3, 4) were fitted to the least squares regression model (columns 5, 6). The Sholl least squares mean values were compared pairwise against the control (column 7). Isomer: 10S,17S-diHDHA

## Results

### TH-Positive Neuron Cell Death Triggered by Rotenone and MPP+ is Prevented by NPD1

**Fig. 1:**
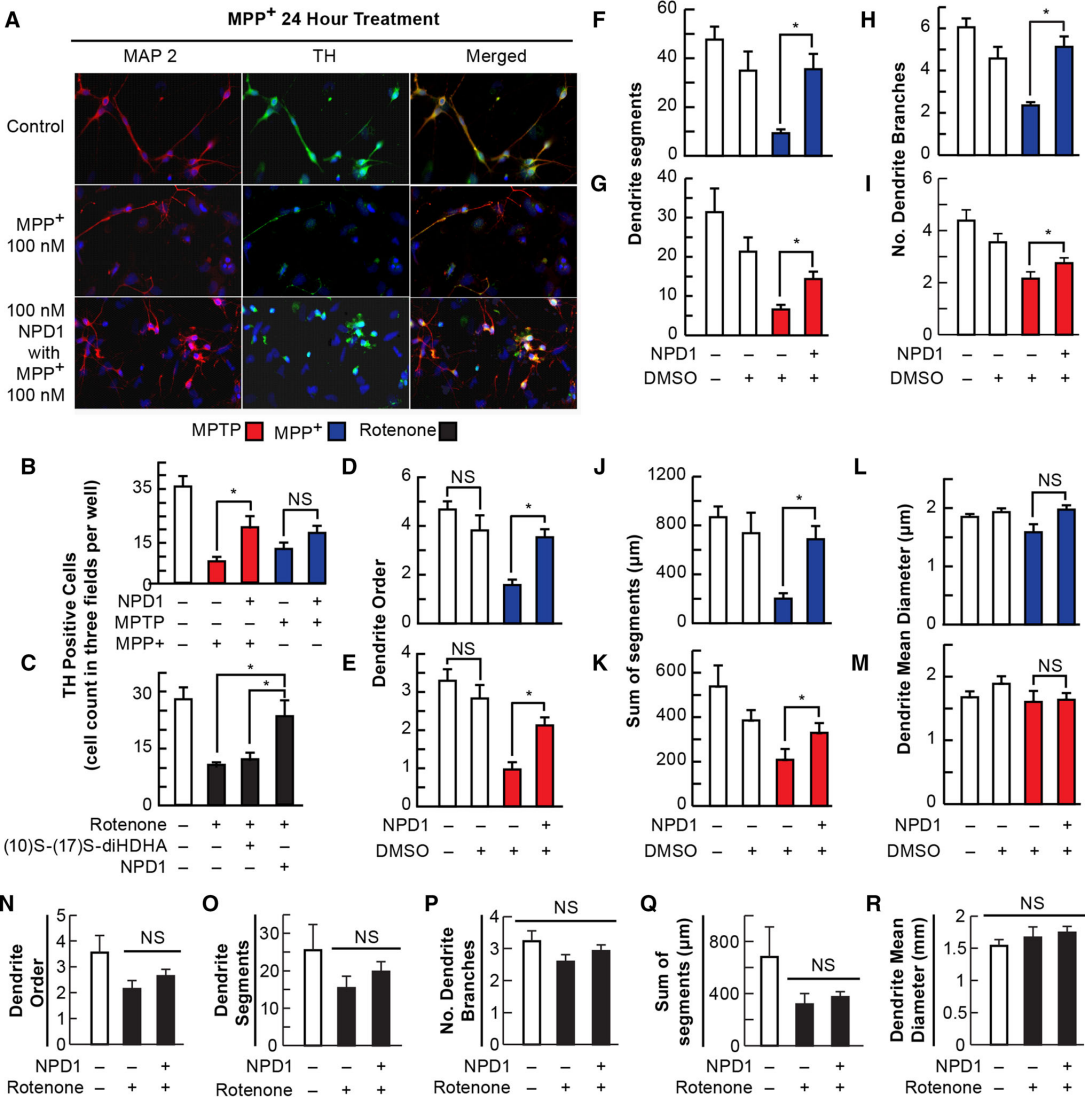
NPD1 promotes survival and prevents dendrite dystrophy of dopaminergic neurons upon addition of MPTP, MPP+, and rotenone. **a**–**c** Neuron survival was measured by a direct count of tyrosine hydroxylase-positive neurons per random field by triplicate per well in at least two wells per condition in two independent experiments. **a** Representative images of mesencephalic neuronal culture neurons stained with MAP2 (*red* *first column*) and tyrosine hydroxylase (*green second column*); nuclei are *blue*. **b**, **c** Tyrosine hydroxylase-positive cells after 24-h 100 µM MPP+ or 100 µM MPTP (**b**) and 100 nM rotenone (**c**) treatment in the presence or absence of 100 nM NPD1 (**b**, **c**) or 10S–17S-diHDHA stereoisomer (**c**). **d**–**m** Neurons that survived MPP+ or MPTP in the presence of NPD1 showed a better dendritic architecture than those that were not treated with NPD1. **d**, **e** Maximum dendritic order, **f**, **g** number of segments, **h**, **i** number of branches, **j**, **k** sum of the length of all the segments per neuron, and **l**, **m** the mean diameter of the dendrites in MPP+- (**d**, **f**, **h**, **j**, **l**) or MPTP- (**e**, **g**, **i**, **k**, **m**) treated cells in the presence or absence of NPD1. DMSO was added as vehicle control. Isolated TH-positive neurons from three random fields per well in two wells per experiment were traced using IMARIS 7. Two experiments were pooled together. **n**–**r** TH-positive neurons that survived 100 nM rotenone treatment for 24 h: **n** maximum dendritic order, **o** number of segments, **p** number of branches, **q** sum of the length of all the segments per neuron, and **r** the mean diameter of the dendrites. Total neurons included for **d**, **f**, **h**, **j**, **l** were control *N* = 22, DMSO *N* = 11, MPP+ *N* = 25, and MPP+ + NPD1 *N* = 37. For **e**, **g**, **i**, **k**, **m**: control *N* = 23, DMSO *N* = 16, MPP+ *N* = 17 and MPP+ + NPD1 *N* = 30. For **n**–**r**, control *N* = 10, rotenone +, rotenone + NPD1 *N* = 33, and rotenone alone *N* = 18. *Bars* represent the mean ± SEM. *P < 0.05, *NS* non-significant *P* value

**Fig. 2:**
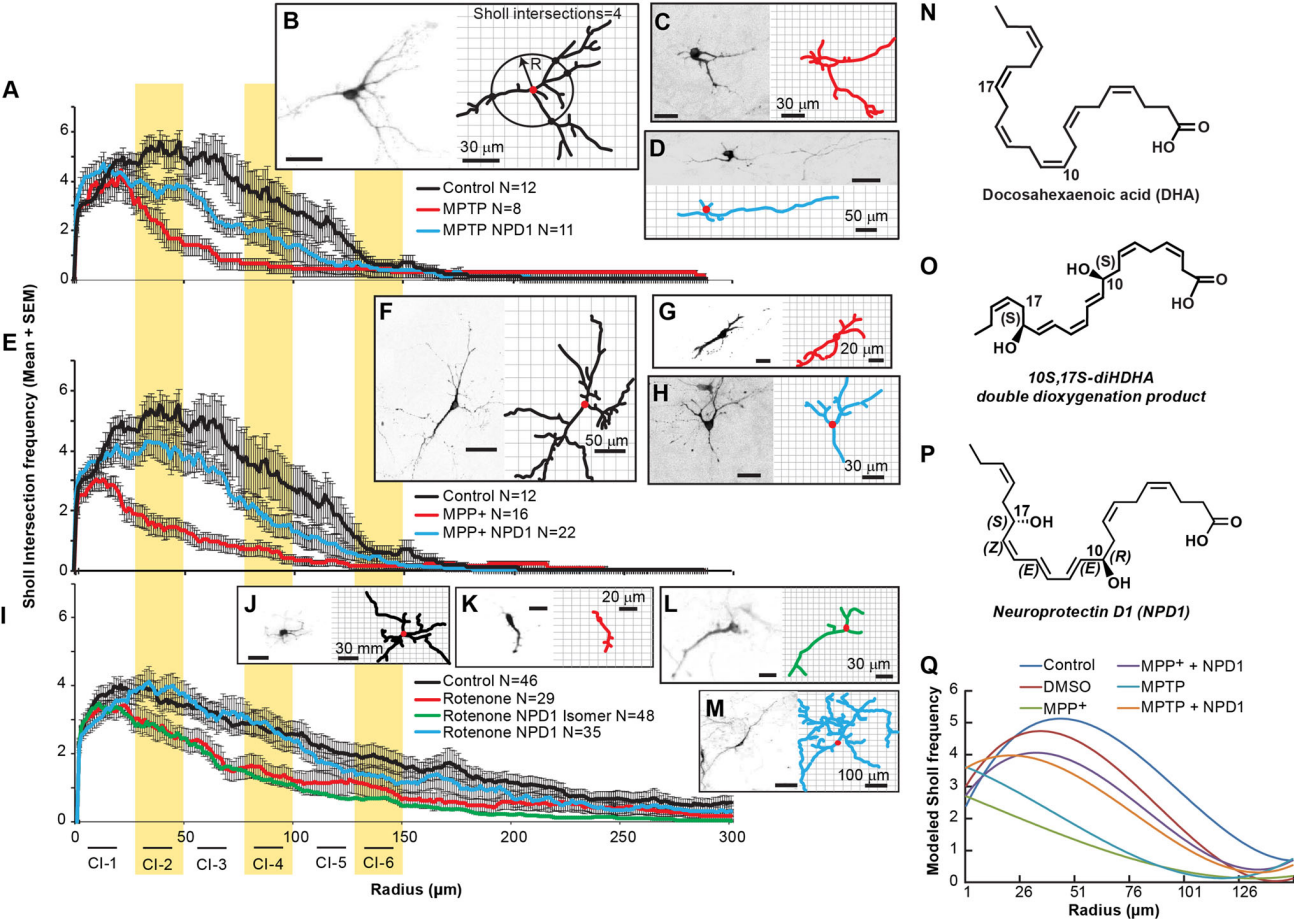
NPD1 prevents disruption of dendritic architecture caused by MPP+, MPTP, or rotenone. The dendritic architecture was assessed using Sholl analysis, which involves calculating the frequency at which the dendritic arbor intersects a series of concentric circles separated by 1 µm using the soma as the center (**b** *right panel*). **a**, **e** Sholl number of intersections (averaged) versus radius of neurons treated with **a** MPTP, **e** MPP+ and **i** rotenone in the presence or absence of NPD1. In all cases, comparisons were made against non-treated neurons (control). The *curves* were fitted to a polynomial regression model using a least squares regression model (*P* < 0.0001); see Supplementary Tables 1–5 and Supplementary Fig. 4. **b**–**d**, **f**–**m** *Right* and *left panels* show a neuron and its corresponding tracings as an output of the IMARIS software, respectively. **b**, **f**, **j** Control neurons or treated with **c**, **d** 100 µm MPTP; **g**, **h** 100 µm MPP+; **k**–**m** rotenone 100 nM; **d**, **h**, **m** plus of NPD1 or **l** its *S*–*S* stereoisomer. The *values* are represented as the mean ± SEM. *NS* non-significant *P* value. **n**, **o** Molecular structure of **n** DHA; **o** 10S-17S-diHDHAstereoisomer and **p** NPD1. **q** Polynomial regression model for MPP+ and MPTP Sholl intersections. The mathematical formulas for the curves plotted are described in Supplementary Table 1. Isomer: 10S,17S-diHDHA

Rotenone and the MPTP metabolite, MPP+, was proposed to induce toxicity primarily by blocking mitochondrial Complex I, which results in the production of superoxide radicals and consequently, oxidative stress (Ramachandiran et al. 2007). To determine the ability of NPD1 to prevent cell death induced by these three compounds, mesencephalic primary-cultured neurons were exposed to 100 nM rotenone, 100 µM MPTP, or 100 µM MPP+ in the presence and absence of NPD1 for 24 h (Fig. 1a–c). MPP+ severely decreased the number of TH-positive neurons from 40.3 ± 7.3 in the control to 7.6 ± 1.6; with the addition of NPD1, the number of surviving cells was raised to 14.4 ± 2.7 (Fig. 1b). MPTP, the precursor of MPP+, also displayed toxicity, promoting the disappearance of 65.7 % of the TH-positive neurons, but this effect was not significantly counteracted by NPD1 (Fig. 1b). On the other hand, rotenone reduced the TH-positive neuronal population 63 %, and NPD1 reversed that effect almost completely (Fig. 1c). The NPD1 stereoisomer (10S,17S-diHDHA) had no effect on TH-positive cell survival, indicating that the protection was specifically induced by the *S*/*R* stereoisomer (Figs. 1c, 2o, p).

### Neurons That Survived with the Addition of NPD1 Showed Better Dendritic Morphology on Average

Mitochondrial damage and depletion leads to neurite retraction or shortening (Cherra et al. 2013). We analyzed the dendritic arbors of surviving TH-positive neurons to evaluate whether NPD1 addition affected neurite integrity. General features were assessed, including maximum dendritic order reached for each TH-positive neuron, number of dendrite segments and branches, the sum of all the segment lengths for each neuron, and the mean for the dendrite diameters. MPTP and MPP+ only altered the parameters related to the length of the dendrites and not related to their thickness, as shown in Fig. 1d–m. Overall, the integrity of the dendritic arbor was best preserved in cells treated with NPD1 in comparison with the ones that survived without NPD1 treatment (Fig. 1d–m). Although more TH-positive neurons survived the rotenone treatment with the addition of NPD1 (Fig. 1c), there was no difference in the maximum dendritic order, the number of segments, or the combined length of all branches and segments when neurons were treated with the lipid mediator (Fig. 1n, o, q). Intriguingly, the number of branches (Fig. 1p) on average did not show any significant changes in rotenone-treated TH-positive neurons when compared to controls, suggesting a different mechanism of toxicity. On the contrary, MPP+ induced a reduction in length and number of branches (Fig. 1d, f, h, j, l), and these effects were reversed almost completely by NPD1. MPTP produced an outcome similar to MPP+, although NPD1 did not provide the same level of improvement (Fig. 1e, g, i, k, m).

## 2D Analysis of Dendritic Branching shows that NPD1 prevents Neurite Retraction at Short to Medium Radii

The Sholl number is the frequency by which neurites intersect in concentric circles drawn around the soma at different radii (Fig. 2b). To further visualize spatially the alterations introduced by MPP+ and its precursor MPTP or rotenone, the total number of Sholl intersections per treatment were averaged and plotted versus the radius (Fig. 2a, e, i). Two-way ANOVA showed significant differences between treatments (Supplementary Tables 2, 3). The Sholl values were fit to a polynomial curve using the least squares model (Supplementary Table 1; Fig. 2q). To assess the distances from the soma at which the differences occurred in the dendritic tree, the curves were broken down into six class intervals containing radii from 0 to 25, 26 to 50, 51 to 75, 76 to 100, 101 to 125, and 126 to 150 µm (Tables 1, 2). Pairwise comparisons between treatments in each interval showed that MPP+ induced a consistent and significant decrease in neurites at all radii and that this effect was totally reversed by NPD1 within the interval from 0 to 25 µm and partially from radius 26 µm or over (Table 1). MPP+- and MPTP-treated neurons showed significant differences, in the presence and absence of NPD1, for radii 0–125 µm (Table 1; Fig. 2a, e). On the other hand, NPD1 stereo-specifically reversed rotenone-induced reduction in the Sholl number at radii 25–100 µm (Fig. 2i; Table 2). Altogether, these results suggest that MPP+ and rotenone exert their toxicity through different mechanisms and that NPD1 halts damage caused by rotenone and MPP+ to TH-positive neurons.

### Terminal Neurites are protected from MPP+ Toxicity by NPD1

**Fig. 3:**
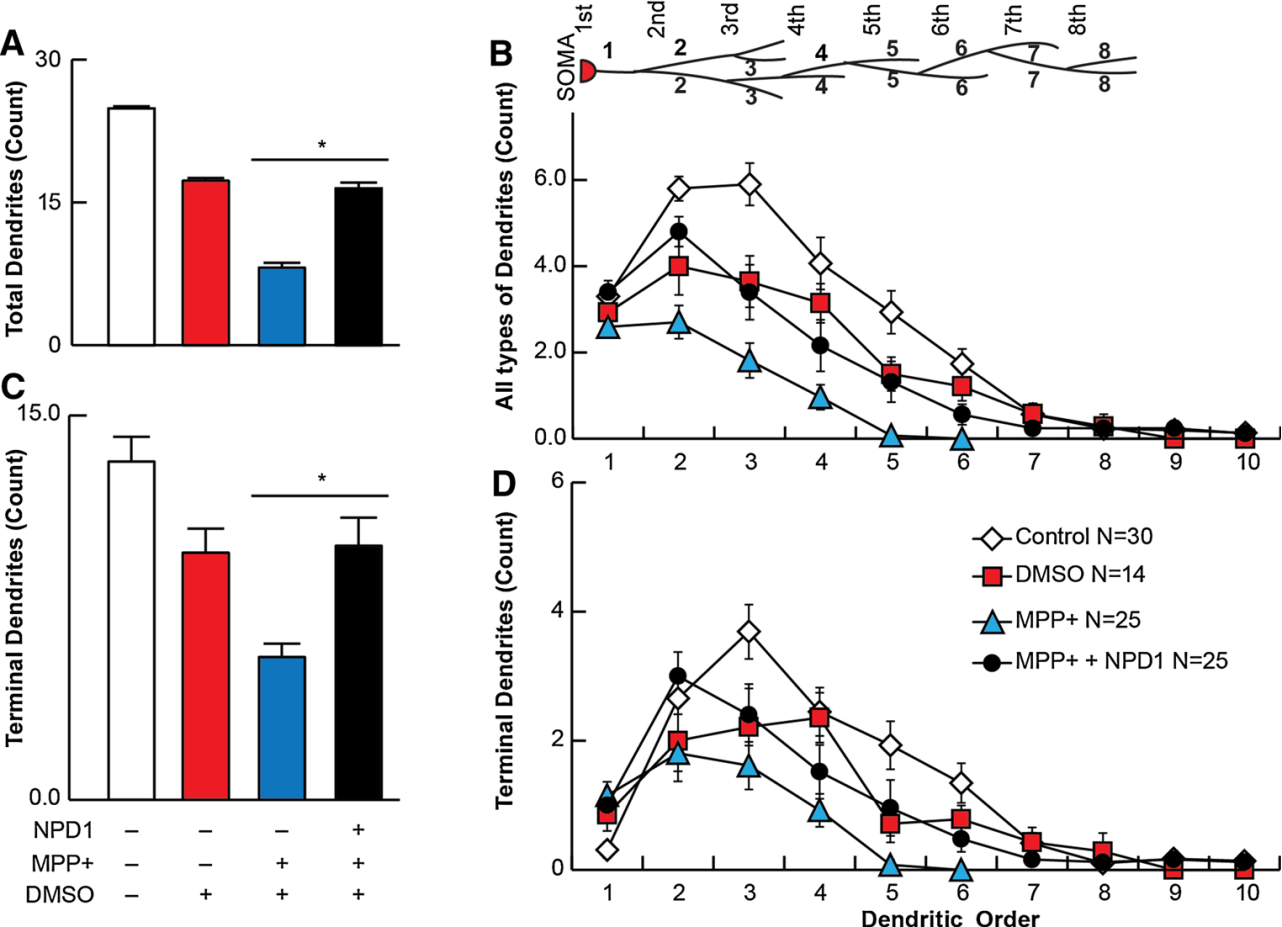
Terminal dendrites are preserved with NPD1 treatment. TH-positive neurons were traced using a NeuronJ plug-in of ImageJ. **a**, **c** Average of the dendritic order. **b**, **d** Average of segments for each dendritic order (**a**, **b**) and terminal dendrites (**c**, **d**) in control, DMSO- or MPP+-treated cells, the latter in the presence or absence of NPD1. The *values* are represented as the mean ± SEM. *P < 0.05. *NS* non-significant *P* value

Total and terminal number of dendrites were plotted versus the dendritic order to assess the location of damage within the dendritic tree. On average, the total dendritic segments were decreased from 24.9 ± 2 in the control to 8.1 ± 1 in neurons treated with MPP+ (Fig. 3a), and the average terminal number of segments followed the same trend (Fig. 3c). The vehicle control DMSO showed a reduction of about 30 % in total segments (Fig. 3a). The number of total and terminal segments started to decline at the dendritic order 2 for the MPP+-treated neurons, and the greatest difference in numbers was reached at dendritic order 3. NPD1 was more effective in protecting dendritic segments at order 2. Following that order, even in the presence of NPD1, the number of total segments was reduced significantly in comparison with the control (Fig. 3b). Terminal branches behaved in a different way. In control cells, a lesser proportion of primary neurites was terminal (Fig. 3c, d). Consistent with the effect of the terminal segments that induced their retraction, the order at which the curve peaked was lower with MPP+, so this was the order at which the values became zero; this means the most abundant terminal neurites were located at order 2, and there were no dendrites at order 6. Once again, NPD1-treated neurons presented dendritic arbor architecture similar in configuration to that of the vehicle, as well as a preserved maximum distal dendrite order.

Altogether, these results suggest that the damage caused by MPP+ induces a diminished arborization, which is a product of terminal neurite retraction. The addition of NPD1 reversed the damage produced by MPP+, bringing the number of dendrites back to the levels of vehicle-treated neurons.

## Discussion

NPD1 is a docosahexaenoic acid derivative shown to be protective in neurons and RPE cells undergoing proteotoxic and oxidative stress damage (Mukherjee et al. 2004; Calandria et al. 2012) as well as in other dysruptions of homeostasis (Bazan et al. 2011). Here we report specific NPD1 effects on the survival and dendrite architecture of TH-positive cells exposed to MPTP, its metabolite MPP+, or rotenone. NPD1 increased the survival of TH-positive cells treated with MPP+ (Fig. 1b) or rotenone (Fig. 1c). MPTP treatment did not reduce the neuron population to the same extent, and NPD1-mediated neuroprotection was not as strong as with the other two toxins (Fig. 1b). Interestingly, MPTP toxicity was not reversed by NPD1 to the same extent as MPP+, suggesting that both actions are executed by different mechanisms. MPTP conversion to MPP+ and release occurs in glial cells (Ransom et al. 1987). It may be possible that this processing of MPTP delays and decreases the amount of MPP+ that reaches neurons, thus causing a weaker but sustained damage compared to the acute insult produced by MPP+. This may explain the differences in TH-positive neurons survival and the protection exerted by NPD1 (Fig. 1b).

Here we report an 81 % disappearance of TH-positive neurons (from 40.3 ± 7.3 in the control to 7.6 ± 1.6 in MPP+-treated neurons). MPP+ selectively damages cells expressing DAT (Holzschuh et al. 2001), and at least 81 % of the TH-positive cells (17–20 % of the total neuronal population) are dopaminergic neurons. Consequently, the majority of cells rescued from death by NPD1 were also dopaminergic. We cannot discount, however, the presence of other catecholaminergic cells. In fact, damage in the noradrenergic system increases dopaminergic neurotoxicity in MPTP-treated primate and MPTP-treated rodent animal models (Fornai et al. 1997); however, there is no clear evidence that non-dopaminergic neurons in culture reacts to MPP+.

DHA decreases caspase-dependent actin cleavage at dendrites when neurons undergo oxidative stress (Calon et al. 2004). Also, under oxidative stress conditions, DHA is converted into NPD1 (Calandria et al. 2009) and the latter decreases caspase activation in neurons (Calandria et al. 2012; Mukherjee et al. 2004).

The neurons surviving MPTP or MPP+ treatments showed, on average, a decrease in dendrite order and number of dendritic segments, as well as reduced branching (Fig. 1d–k). Rotenone affected the dendritic order and number of segments (Fig. 1n, o, q) but did not affect the number of branches (Fig. 1p). Accordingly, the extent and pattern of the damage displayed in the assessment of Sholl intersections differed significantly from MPTP/MPP+ and rotenone, suggesting specific mechanisms of action for both types of toxins.

MPP+, a metabolite of MPTP, induces damage in SNpc dopaminergic neurons. Although it is not known why this metabolite prefers SNpc neurons specifically, it was proposed that MPP+ is internalized by neurons through the dopamine transporter DAT and that it may induce oxidation of intracellular dopamine, which confers dopaminergic neuronal specificity to the toxin (Holzschuh et al. 2001). It was proposed that MPP+ inhibits mitochondrial Complex I, enhancing the production of reactive oxygen species (ROS) (Cleeter et al. 1992) and inducing a mild depletion in ATP (Chan et al. 1991), features observed in PD postmortem brains (Parker et al. 1989; Schapira et al. 1990; Keeney et al. 2006).

Other lines of evidence contradict the Complex I hypothesis. For instance, midbrain mesencephalic-cultured dopaminergic neurons that lack Ndufs4, a key assembly subunit in Complex I and thus a functional Complex I, have survived and shown normal morphology (Choi et al. 2008). These neurons displayed more sensitivity to rotenone and MPP+ than the Ndufs4+/+ dopaminergic neurons. Following this line of thought, it was recently proposed that microtubule dysfunction may be the mechanism responsible for the rotenone effect on dopaminergic cells, and the lack of functional Complex I may potentiate this toxicity (Choi et al. 2011). It also was shown that MPP+ (Cappelletti et al. 2005; Cartelli et al. 2010) and rotenone (Choi et al. 2011) have an effect on the cytoskeleton that may provide an explanation for the shrinkage of the dendritic tree. Since microtubular dysfunction affects neuronal survival in different ways, it may be an early indicator of neurodegeneration. For instance, autophagy, a microtubular-dependent mechanism during stress conditions (Mackeh et al. 2013), clears damaged mitochondria to prevent toxicity. This process is called mitophagy and is regulated by PINK (Ashrafi and Schwarz 2013), involved in hereditary PD (Valente et al. 2004). Furthermore, autophagy was found to be compromised in conditional ATG7 knockout mice, with a subsequent decrease of dopaminergic neurons in the SNpc and an accumulation of α-synuclein and LRRK2 (Friedman et al. 2012). Our results indicate that terminal dendrites are compromised in MPP+-surviving TH-positive neurons (Fig. 3). Dendritic dystrophy, a reduction in length and complexity of dendrites, is a common feature of neurodegeneration, most often observed in PD (Friedman et al. 2012). Although in our cellular model, no morphological signals of initial dendrotoxicity, such as neurite beading, were observed after 24 h of treatment, the disappearance of terminal segments (Figs. 1d–g, j–k, n–o, q, 3) and diminished branching occurred (Figs. 1h–i, 2a, e, i) in response to MPTP, MPP+ and rotenone. In the Drosophila’s larval multidendritic neuron model, dendrite degeneration is an active programmed process that can take place within the first 24 h of toxic exposure (Tao and Rolls 2011). This timing may explain the absence of dendritic beading observed.

Protein phosphate 2A (PP2A) is a master regulator of the phosphorylation/dephosphorylation processes, including microtubule stabilization/destabilization. Recently, NPD1 modulates PP2A activity (Antony et al. 2010; Calandria et al. 2012). Furthermore, PP2A was involved in the microtubular dysfunction related to tauopathies present in Alzheimer’s disease (Sontag et al. 2012) and also found in postmortem PD brains (Wills et al. 2010). Therefore, the pro-survival activity of NPD1 also may be preventing dendrite retraction.

Taken together, our results suggest that NPD1 promotes survival and preservation of the dendritic tree in dopaminergic neurons undergoing MPP+ or rotenone toxicity by preventing microtubular dysfunction and oxidative stress-driven cell death. The mechanisms by which NPD1 counteracted TH-positive apoptosis and dendrite dystrophy triggered by MPP+ and rotenone may involve the activation of PP2A and the inhibition of effector caspases. These findings lay the groundwork for understanding how NPD1 signaling may be manipulated to improve the survival of SNpc dopaminergic cells in preclinical stages of PD and to prevent the progression of neurodegeneration. In future studies, efforts will be made to identify the therapeutic targets using this information. In this way, it may be possible to promote NPD1 signaling at early stages in at-risk populations to reduce the incidence, delay the onset, or lessen the severity of the symptoms later in life by preventing—at least to some extent—dopaminergic cells from dying.

## Electronic supplementary material

Below is the link to the electronic supplementary material.

Supplementary material 1 (RTF 34743 kb)

Supplementary material 2 (DOCX 16 kb)
