# Supplementary material for: The Docosanoid Neuroprotectin D1 Induces TH-Positive Neuronal Survival in a Cellular Model of Parkinson’s Disease
Source: Cell Mol Neurobiol. 2015 Jun 6;35(8):1127–36. doi: 10.1007/s10571-015-0206-6 (PMC4602058; doi:10.1007/s10571-015-0206-6)
Supplement: Supplementary file 1 — Supplementary material 1 (RTF 34743 kb) [file 10571_2015_206_MOESM1_ESM.rtf]

Supplementary Figure Legends


Supplementary Figure 1: Optimization of enriched dopaminergic neuronal culture. Three fields obtained from a culture without (A) or with (B) one-time addition of 10 ng/ml GDNF, 2 ng/ml TGF-b and 50 µM dcAMP, following a variation of the protocol described by Sun et al., 2004 in Cell Biology International 28:323-325. MAP2 (green), TH (red), nuclei (blue).


Supplementary Figure 2:  Time optimization for MPTP treatment. Fields of enriched dopaminergic neuronal culture at 24 h (A) and 48 h (B) when cells were treated with 100 µM MPTP in the presence or absence of 100 nM NPD1. b III tubulin (red), TH (green), nuclei (blue).


Supplementary Figure 3:  Time optimization for rotenone treatment. Fields of enriched dopaminergic neuronal culture at 24 h (A) and 48 h (B) when cells were treated with 100 nM rotenone in the presence or absence of 100 nM NPD1. bIII tubulin (red), TH (green), nuclei (blue).


Supp Fig 1


Supp Fig 2


Supp Fig 3
