# Supplementary material for: The Docosanoid Neuroprotectin D1 Induces TH-Positive Neuronal Survival in a Cellular Model of Parkinson’s Disease
Source: Cell Mol Neurobiol. 2015 Jun 6;35(8):1127–36. doi: 10.1007/s10571-015-0206-6 (PMC4602058; doi:10.1007/s10571-015-0206-6)
Supplement: Supplementary file 2 — Supplementary material 2 (DOCX 16 kb) [file 10571_2015_206_MOESM2_ESM.docx]

**Supplementary Tables**

Supplementary Table 1: Mathematical expressions of the curves plotted in Figure 2Q. Polynomial functions obtained using least squares fitting for the Sholl intersection data plotted in Figure 2A, E and I, depicted for control, vehicle (DMSO) and MPP+ or MPTP in the presence or absence of NPD1. In the right column, the R-squared for each curve shows the proportion of the observations that the model is able to explain.

| **Treatment** | Mathematical function (least squares) | R-squared |
| --- | --- | --- |
| Control | $Sholl\#=\left( 2.24839\pm0.19821 \right)+\left( 0.14469 \pm0.01149 \right)r-\left( 0.00212 \pm0.00017815 \right)r^{2}+(0.00000724 \pm0.00000078)r^{3}$ * | 0.3329 |
| DMSO | $Sholl\#=\left( 2.90504\pm0.155577 \right)+\left( 0.11406 \pm0.00902 \right)r-\left( 0.00203 \pm0.00014005 \right)r^{2}+(0.00000764 \pm0.00000061)r^{3}$ * | 0.5673 |
| MPP+ | $Sholl\#=\left( 2.73168\pm0.07661 \right)-\left( 0.02809 \pm0.0444 \right)r-\left( 0.00001986 \pm0.0000689 \right)r^{2}+\left( 0.000000632 \pm0.0000003 \right)r^{3}$ * | 0.4214 |
| MPP+ + NPD1 | $Sholl\#=\left( 2.64220 \pm0.11602 \right)+\left( 0.09371\pm0.00672 \right)r-\left( 0.00177 \pm0.000104 \right)r^{2}+(0.00000706 \pm0.000000457)r^{3}$ * | 0.3984 |
| MPTP | $Sholl\#=\left( 3.64562\pm0.13019 \right)-\left( 0.02749 \pm0.00754 \right)r-\left( 0.0002893 \pm0.000117 \right)r^{2}+(0.00000229 \pm0.00000051)r^{3}$ * | 0.4928 |
| MPTP + NPD1 | $Sholl\#=\left( 2.24839\pm0.19821 \right)+\left( 0.14469 \pm0.01149 \right)r-\left( 0.00212 \pm0.00017815 \right)r^{2}+(0.00000724 \pm0.00000078)r^{3}$ * | 0.4796 |

*ANOVA: P < 0.0001

Supplementary Table 2: Sholl number mean and standard error of the mean (Columns 2 and 3) under the least squares model (Columns 4 and 5) for neurons surviving MPTP and MPP+ 24 h treatment in the presence or absence of 100 nM NPD1.

| Treatment | Sholl Mean | Standard Error of the Mean | Least Squares Mean | Standard Error of Least Squares Mean |
| --- | --- | --- | --- | --- |
| Control | 3.285863 | 0.061667 | 3.28918981 | 0.02959357 |
| DMSO | 2.738014 | 0.059916 | 2.71629630 | 0.02949135 |
| MPP+ | 1.043664 | 0.026059 | 1.01916667 | 0.02949135 |
| MPP+ + NPD1 | 2.383250 | 0.048697 | 2.32484848 | 0.02949135 |
| MPTP | 1.391267 | 0.047278 | 1.36083333 | 0.02949135 |
| MPTP NPD1 | 2.298257 | 0.048697 | 2.24666667 | 0.02949135 |

ANOVA p< 0.0001

Supplementary Table 3: Sholl number mean and standard error of the mean (Columns 2 and 3) under the least squares model (Columns 4 and 5) for neurons surviving rotenone 24 h treatment in the presence or absence of 100 nM NPD1.

| Treatment | Sholl Mean | Standard Error of the Mean | Least Squares Mean | Standard Error of Least Squares Mean |
| --- | --- | --- | --- | --- |
| Control | 2.862174 | 0.025493 | 2.862174 | 0.02228427 |
| Rotenone + NPD1 | 2.697524 | 0.030424 | 2.697524 | 0.02554720 |
| Rotenone | 1.909655 | 0.029116 | 1.909655 | 0.02806585 |
| Rotenone Isomer | 1.741389 | 0.021636 | 1.771884 | 0.02228427 |

ANOVA p<0.0001
